# National TB cohort review evaluation: insights for control strategies in low-incidence settings

**DOI:** 10.5588/ijtldopen.26.0678

**Published:** 2026-06-15

**Authors:** T.D. Barry, J. Underwood, M. Backx, M. Brouns, A. Story, G. Lowe, L. Weeks, Y. Hester, P. Lloyd, R. Bunce, L. Johnstone, P. Pallmann, E. Thomas-Jones, N. Murray, K. Jones, D.R. Thomas, G. Ahern, K. Metters, J. Smith, S.M. Barry

**Affiliations:** 1 Division of Population Medicine, Cardiff University, Cardiff, UK;; 2 Communicable Disease Surveillance Centre, Public Health Wales, Cardiff, UK;; 3 Division of Infection and Immunity, Cardiff University, Cardiff, UK;; 4 Department of Infectious Diseases, Cardiff and Vale University Health Board, Cardiff, UK;; 5 Department of Respiratory Medicine, Aneurin Bevan University Health Board, Abergavenny, UK;; 6 UCL Collaborative Centre for Inclusion Health, University College London, London, UK;; 7 Department of Respiratory Medicine, Cardiff and Vale University Health Board, Cardiff, UK;; 8 Department of Respiratory Medicine, Betsi Cadwaladr University Health Board, Wrexham, UK;; 9 Department of Respiratory Medicine, Swansea Bay University Health Board, Swansea, UK;; 10 Centre for Trials Research, Cardiff University, Cardiff, UK;; 11 Department of Respiratory Medicine, Cwm Taf University Health Board, Llantrisant, UK;; 12 Department of Respiratory Medicine, Hywel Dda University Health Board, Carmarthen, UK;; 13 Cardiff University School of Medicine, Cardiff, UK.

**Keywords:** tuberculosis, cohort review, control programme, surveillance, low incidence

## Abstract

**BACKGROUND:**

TB cohort review is a structured clinical audit of case management and epidemiological data. In 2012, Wales (UK) implemented a national cohort review programme to improve clinical outcomes and enhance public health surveillance.

**METHODS:**

Retrospective population-based cohort analysis of care quality outcomes for all notified TB cases in Wales (2012–2022). Adjusted regression modelling assessed temporal trends post-implementation and compared outcomes of cases presented at TB cohort review with those not presented.

**RESULTS:**

Of the 1,175 notified TB cases over the study period, 985 (83.1%) were presented at cohort review. Many case management outcomes achieved near-target thresholds. Adherence support was increasingly targeted at index cases with social complexity and higher transmission risk, alongside substantial improvements in the completeness of surveillance data. Persistent programmatic challenges in contact identification and assessment were identified.

**CONCLUSION:**

As countries approach pre-elimination thresholds, TB services must ensure rigorous surveillance data collection and targeting of limited resources to populations at greatest risk of transmission and non-completion of treatment. Since implementing a national TB cohort review in Wales, there have been significant post-implementation improvements in care outcomes. This provides a robust, resource-efficient framework for TB control that could be applied to other similarly resourced low-incidence countries.

The incidence of TB in the UK and many European countries has continued to decline over recent decades. Wales has an incidence of 3.0 per 100,000 population and, like many other European countries, is classified as low-incidence (<10 cases per 100,000 population).^1–3^ However, the rate of decline has slowed, and TB epidemiology is shifting, with cases concentrated in migrants from high-incidence countries and socially marginalised groups. This presents new challenges that TB control programmes must adapt to.^4^

TB cohort review (TBCR) is a systematic approach to case management and surveillance that involves periodic reviews of notified TB cases by multidisciplinary health care teams specialised in TB management. Meetings comprise specialist TB physicians and nurses, non-specialist clinical staff, health protection teams, and an external TB expert. Central to CR are predefined national outcome targets and comprehensive data-collection protocols, which facilitate service evaluation and quality-improvement initiatives.^5^ Wales implemented TBCR in 2012, partly in response to a large outbreak in 2010, which highlighted the inadequate framework for dealing with TB outbreaks in Wales.^6^ The programme operates as a routine national process targeting all notified TB disease cases rather than being limited to specific subgroups. Despite the adoption of TBCR in other low-incidence settings such as London and New York,^7,8^ there is limited evidence on its long-term impact when implemented nationally.

## METHODS

We conducted a retrospective population-based cohort study using routinely collected TB surveillance data to examine temporal changes in case management and contact tracing outcomes among all notified TB cases in Wales from 2012 to 2022. We asked three research questions: i) Did case management and contact tracing outcomes progress towards national targets over the study period? ii) Were observed temporal changes explained by changes in patient demographics and clinical characteristics? iii) Did progress towards targets differ between cases presented and not presented at TBCR meetings?

### Study population

All notified cases of TB disease in Wales between 2012 and 2022 were included in the study. No exclusions were applied.

### Data sources and variables

Multidisciplinary teams manage cases across six of the seven local health boards (LHBs) in Wales. Cases in Powys Teaching HB are managed by teams in adjacent LHBs or in England. TB teams collected standardised demographic, clinical, and programme-monitoring data using Enhanced TB Surveillance forms from 2012 to 2021 and National TB Surveillance System forms from 2022 onwards.

Case management outcome variables were HIV testing offered, use of enhanced case management (ECM; intensified follow-up and support when risk of non-adherence is identified), requirement and offer of directly observed therapy (DOT), sputum culture confirmation, and treatment completion. Contact tracing outcomes were the number of close contacts identified, clinically assessed, and completing TB preventive treatment (TPT). The primary predictor was the year of case notification. Adjustment covariates included presentation status (whether the case was presented at TBCR), age (years), sex at birth, migration status (UK-born/non-UK-born), TB site (pulmonary with/without extra-pulmonary vs. extra-pulmonary only), and ≥1 self-reported social risk factor (history of homelessness, imprisonment, or substance/alcohol misuse). Contact tracing outcomes were additionally adjusted for chest X-ray cavities and sputum smear positivity.

### Statistical analysis

Categorical variables were summarised as counts and percentages, and continuous variables as medians and interquartile ranges. Calendar years were grouped into three ordered time periods to minimise small cell counts and highlight temporal trends. Trends were evaluated using Cochran–Armitage tests for categorical variables across periods and Jonckheere–Terpstra tests for continuous variables. Regression modelling examined temporal trends using four model specifications per outcome: i) unadjusted trend, ii) unadjusted interaction model (year × presentation), iii) adjusted trend, and iv) adjusted interaction model. Interaction terms were tested to assess whether trends differed by presentation status. Binary case management outcomes were examined using binomial logistic regression, contacts identified using negative binomial regression to account for overdispersion, and contact assessment and TPT completion using quasibinomial regression models with explicit denominators to estimate proportions. Non-linear associations between continuous variables and exposure were tested using likelihood-ratio tests and modelled using natural cubic splines (3 degrees of freedom).

### Missing data

Compatibility with a missing-at-random (MAR) assumption was assessed by modelling predictors of missingness using binomial logistic regression and applying Little’s missing-completely-at-random (MCAR) test.^9^ Multiple imputation by chained equations (40 imputations) was performed using the *mice* R package (version 3.17.0).^10^ Convergence and plausibility of imputed values were checked using trace and density plots. The fraction of missing information (FMI) was reported per outcome. Contact data were not imputed as we could not reliably distinguish missing data from true zeros.

### Sensitivity analyses

We compared multiply imputed estimates with those from complete-case analysis (CCA) and assessed the robustness of the estimates to imputation specification. All statistical analyses were conducted in R version 4.3.3.^11^ A two-sided *P* < 0.05 was considered significant. Reporting followed STROBE (Strengthening the Reporting of Observational Studies in Epidemiology) guidelines.^12^

### Ethical statement

This study received ethical approval from the UK Central Bristol Research Ethics Committee (23/SW/0089, IRAS 321836). Surveillance case numbers ensured participant confidentiality.

## RESULTS

Between 2012 and 2022, 1,175 cases of TB disease were notified in Wales, of which 985 (83.1%) were presented at TBCR (Table 1). Presentation at TBCR increased over time (*P* = 0.001), associated with calendar year and LHB (both *P* < 0.001), but not case complexity (*P* = 0.267; Supplementary Data Table S1). Cases were concentrated in urban South Wales, particularly Cardiff and Vale (29.4%) and Aneurin Bevan (21.6%). Most occurred in adults aged 20–49 years (58.4%) and non-UK-born persons (59.1%). The proportion of non-UK-born cases resident in the UK for ≥10 years increased (47.4% by 2019–2022, *P* < 0.001), as did interferon-gamma release assay (IGRA) arrival screening (27.5%, *P* = 0.007). Social complexity increased, particularly for UK-born cases (≥2 social risk factors 6.8%–21.2%, *P* < 0.001). Participant flow is shown in Supplementary Data Figure S1.

**Table 1.** Demographic and clinical characteristics of notified TB cases in Wales, 2012–2022.

Characteristic	Total		2012–2015		2016–2018		2019–2022		P
	n/N	%	n/N	%	n/N	%	n/N	%	
Total notified TB cases	1,175	100.0	525	44.6	311	26.4	339	28.9	–
Presented at CR	985/1,175	83.1	413/525	78.7	280/311	90.0	292/339	86.1	**0.001**
Demographics									
Age (years)									
Median (IQR)	42 (29–58)	–	41 (28–58)	–	43 (31–60)	–	41 (30–55)	–	0.539
<20	70/1,175	6.0	28/525	5.3	22/311	7.1	20/339	5.9	0.652
20–49	686/1,175	58.4	307/525	58.5	174/311	55.9	205/339	60.5	0.643
≥50	419/1,175	35.7	190/525	36.2	115/311	37.0	114/339	33.6	0.485
Male sex	727/1,175	61.9	332/525	63.2	191/311	61.4	204/339	60.2	0.357
Social risk factors									
≥1	165/1,129	14.6	56/502	11.2	47/301	15.6	62/326	19.0	**0.001**
≥2	90/1,129	8.0	23/502	4.6	32/301	10.6	35/326	10.7	**0.001**
Any missing	203/1,175	17.3	106/525	20.2	54/301	17.4	43/339	12.7	**<0.001**
UK-born (≥2)	62/458	13.5	14/205	6.8	24/140	17.1	24/113	21.2	**<0.001**
Migration status									
Non-UK-born	694/1,167	59.4	307/519	59.2	164/309	53.1	223/339	65.8	0.103
<2 years in UK	115/588	19.6	45/256	17.6	31/142	21.8	39/190	20.5	0.406
≥10 years in UK	215/588	36.6	75/256	29.3	50/142	35.2	90/190	47.4	**<0.001**
Missing	32/694	4.6	19/307	6.2	9/164	5.5	4/223	1.8	**0.020**
IGRA arrival screening	75/381	19.7	20/142	14.1	22/119	18.5	33/120	27.5	**0.007**
Missing	313/694	45.1	165/307	53.7	45/164	27.4	103/223	46.2	**0.034**
Local health board									
Aneurin Bevan	254/1,175	21.6	101/525	19.2	67/311	21.5	86/339	25.4	**0.034**
Betsi Cadwaladr	182/1,175	15.4	80/525	15.2	49/311	15.8	53/339	15.6	0.863
Cardiff and Vale	346/1,175	29.4	148/525	28.2	96/311	30.9	102/339	30.1	0.505
Cwm Taf Morgannwg	113/1,175	9.6	43/525	8.2	30/311	9.6	40/339	11.8	0.080
Hywel Dda	99/1,175	8.4	59/525	11.2	24/311	7.7	16/339	4.7	**0.001**
Swansea Bay	130/1,175	11.1	65/525	12.4	34/311	10.9	31/339	9.1	0.139
Outside Wales	51/1,175	4.3	29/525	5.5	11/311	3.5	11/339	3.2	0.092
Clinical									
Referral to assessment, median (IQR)	3 (0–12)	–	2 (0–10)	–	3 (0–13)	–	4 (1–14)	–	**0.001**
Missing	388/1,175	33.0	178/525	33.9	65/311	20.9	145/339	42.8	**0.036**
PTB ± EPTB	713/1,175	60.7	292/525	55.6	204/311	65.6	217/339	64.0	**0.007**
Sputum culture positive	267/343	77.4	12/12	100.0	116/139	83.5	139/192	72.4	**0.003**
Missing	414/713	58.1	280/292	95.9	78/204	38.2	56/217	25.8	**<0.001**
Sputum smear positive	296/520	41.5	137/212	64.6	76/155	49.0	83/153	54.2	**0.029**
Missing	193/713	27.1	80/292	27.4	49/204	24.0	64/217	29.5	0.665
MDR	10/1,175	0.9	4/431	0.9	4/283	1.4	2/304	0.7	0.776
Case management									
HIV testing offered (target 100%)	939/1,016	92.3	349/404	86.4	280/292	95.9	310/320	96.6	**<0.001**
Missing	138/1,175	11.7	111/525	21.2	14/311	4.5	13/339	3.8	**<0.001**
Enhanced case management	385/842	45.7	124/301	41.2	117/258	45.3	144/283	50.9	**0.019**
Missing	333/1,175	28.3	224/525	42.7	53/311	17.0	56/339	16.5	**<0.001**
Requiring DOT	201/926	21.7	50/349	14.3	62/263	23.6	89/314	28.3	**<0.001**
Missing	249/1,175	21.2	176/525	33.5	48/311	15.4	25/339	7.4	**<0.001**
Offered DOT (target 100%)	178/201	96.7	38/41	92.7	54/56	96.4	86/87	98.9	0.068
Missing	17/201	8.5	9/50	18.0	6/62	9.7	2/89	2.2	**<0.001**
Treatment completed (target ≥85%)	990/1,175	84.3	438/508	86.2	257/304	84.5	295/328	89.9	0.166
Lost to follow-up	41/1,175	3.6	21/508	4.1	11/304	3.6	9/328	2.7	0.296
Missing	35/1,175	3.1	17/508	3.2	7/304	2.3	11/328	3.2	0.925
Contact tracing									
≥1 contact identified									
All cases	828/1,175	70.4	344/525	65.5	227/311	73.0	257/339	75.8	**<0.001**
PTB ± EP	537/713	75.3	200/292	68.5	167/204	81.9	170/217	78.3	**0.006**
Smear positive (target ≥95%)	248/296	83.7	116/137	84.7	66/76	86.8	66/83	79.5	0.373
≥5 contacts identified									
All cases	348/1,175	29.6	128/525	24.4	109/311	35.0	111/339	32.7	**0.004**
PTB ± EP	276/713	38.7	96/292	32.9	93/204	45.6	87/217	40.1	0.067
Smear positive (target ≥80%)	143/296	48.3	62/137	45.3	42/76	55.3	39/83	47.0	0.670
Assessed (target ≥90%)	4,914/6,229	79.0	1831/2,217	82.6	1,407/1744	80.7	1,676/2,268	73.9	**<0.001**
TPT completion	391/602	65.0	109/174	62.6	100/180	55.6	182/248	73.4	**0.010**

Descriptive statistics derived from complete-case data. Significant *P* values (*P* < 0.05) shown in bold. Missing data categories shown only when ≥3% of cases had missing values for that variable. *P* values derived from Cochran–Armitage tests for temporal trends in categorical variables and Jonckheere–Terpstra tests for continuous variables. Social risk factors include any self-reported history of homelessness, prison, substance use disorders, or alcohol misuse.

CR = cohort review; IQR = interquartile range; UK = United Kingdom; IGRA = interferon-gamma release assay; PTB = pulmonary TB; EPTB = extra-pulmonary TB; MDR = multidrug-resistant; DOT = directly observed therapy; TPT = TB preventive treatment.

Progress towards national outcome targets varied. HIV testing increased from 86.4% to 96.6% (*P* < 0.001). ECM use rose from 41.2% to 50.9% (*P* = 0.019), and cases judged to require DOT doubled (14.3%–28.3%, *P* < 0.001). Those offered DOT increased from 92.7% to 98.9% (*P* = 0.068). Treatment completion remained high at 84.3% (*P* = 0.166), with 3.6% lost to follow-up. Median time from referral to assessment increased from 2 to 4 days (*P* = 0.001).

For all TB cases, contact identification improved (≥1 contact 65.5%–75.8%, *P* < 0.001 and ≥5 contacts, 24.4%–32.7%, *P* = 0.004). In sputum smear–positive cases, contact identification did not improve (83.7% and 48.3% overall, both *P* > 0.05). Contact assessment declined (82.6%–73.9%, *P* < 0.001), while TPT completion increased (62.6%–73.4%, *P* = 0.010).

After adjustment, most temporal associations were attenuated (Table 2). Only HIV testing and DOT requirement remained significant (adjusted odds ratios [aORs] 1.28 and 1.07, *P* < 0.001 and *P* = 0.040). ECM and DOT were increasingly targeted at social complexity (aORs 5.71 and 11.42, both *P* < 0.001), pulmonary TB (aORs 2.38 and 2.31, both *P* < 0.001), and adults aged <20 years (Supplementary Data Table S2; Supplementary Data Figure S2B). The increase in DOT requirement was attenuated in cases not presented at TBCR (interaction aOR 0.74, *P* = 0.043). HIV testing peaked in middle-aged adults but declined at extremes of age, while treatment completion rates declined sharply in cases aged >65 years (Supplementary Data Figure S2A and S2D).

**Table 2.** Temporal trends in TB cohort review outcomes in Wales, stratified by presentation status, 2012–2022.

Case management outcomes	Eligible cases (N)	Overall temporal trend, aOR (95% CI)	*P* value	Eligible cases (NP|P)	Interaction effect, aOR (95% CI)	*P* value
HIV testing offered	1,017	1.28 (1.17–1.41)	<0.001	85/932	1.01 (0.75–1.35)	0.953
ECM usage	1,175	1.03 (0.98–1.08)	0.283	190/985	0.95 (0.75–1.21)	0.676
DOT required	1,175	1.07 (1.01–1.15)	0.043	190/985	0.74 (0.57–0.97)	0.027
DOT offered	201	1.22 (0.87–1.70)	0.253	12/189	0.82 (0.42–1.60)	0.564
PTB culture confirmation	713	1.02 (0.91–1.13)	0.772	113/600	1.15 (0.57–2.33)	0.698
Treatment completion	1,175	1.06 (0.99–1.12)	0.073	190/985	0.96 (0.82–1.14)	0.663
Data completeness	1,175	1.29 (1.26–1.32)	<0.001	190/985	0.89 (0.85–0.93)	<0.001
Contact tracing outcomes						
Contacts identified (IRR)	1,175	Non-linear, see Supplementary Data Figure S3A	LRT *P* < 0.001	190/985	1.00 (0.93–1.08)	0.901
Contacts assessed	828	Non-linear, see Supplementary Data Figure S3B	LRT *P* < 0.001	44/784	1.08 (0.86–1.35)	0.525
TPT completion	270	1.03 (0.95–1.11)	0.474	15/255	1.26 (0.90–1.75)	0.178

Temporal trends in TB case management and contact tracing outcomes, stratified by presentation status at cohort review. Models adjusted for age, sex, social risk factors (≥1 of homelessness, prison, substance/alcohol misuse), TB site, and UK birth status. Case management outcomes: logistic regression with multiply imputed data. Contact identification: negative binomial regression. Contacts assessed and TPT completion: quasibinomial regression with explicit denominators. Temporal linearity tested using LRT, and natural cubic splines (3df) fitted where non-linearity detected. Overall temporal trend (aOR/IRR): adjusted odds/incidence rate ratio per year for all cases. Interaction effect: differential trend for not-presented (NP) versus presented (P) cases at cohort review. Data completeness refers only to case management outcomes above. Treatment completion involves all notified cases, regardless of drug susceptibility.

CI = confidence interval; ECM = enhanced case management; DOT = directly observed therapy; PTB = pulmonary TB; TPT = TB preventive treatment; IRR = incidence rate ratio; LRT = likelihood-ratio test.

Contact identification showed a non-linear temporal trend, with a mid-study decline followed by an increase from 2019 onwards (Supplementary Data Figure S3A). Fewer contacts were identified for male and non-UK-born cases, but more for pulmonary TB, cavitary disease, and smear-positive cases (Supplementary Data Table S3). Contact assessment also showed a non-linear trend, with fluctuations followed by sustained decline after 2018 (Supplementary Data Figure S3B). Older index cases and those with social risk factors were less likely to have contacts assessed, whereas index males and cavitary disease were associated with higher odds of assessment. TPT completion showed no temporal trend (aOR 1.03, *P* = 0.474), although contacts of smear-positive index cases had higher odds of completion. Trends did not differ by presentation at TBCR, but non-presented cases were substantially less likely to have contacts identified (incidence rate ratio 0.28, *P* < 0.001).

### Missing data and sensitivity analyses

Missing data decreased substantially over the study period (Supplementary Data Table S4), with mean missingness falling from 24.9% (2012–2015) to 7.3% (2019–2022). Overall data completeness improved by 41.8 percentage points (OR 1.29 per year, *P* < 0.001). Improvements were greater for cases presented at TBCR (interaction OR 0.89, *P* < 0.001), and for HIV testing, ECM use, and DOT recording (all *P* < 0.001; Supplementary Data Table S5). Predictors of missingness included year, presentation status, LHB, and disease site (all *P* < 0.001), plausibly supporting an MAR mechanism (Supplementary Data Table S5). Little’s test rejected the null hypothesis of MCAR (*P* < 0.001). MICE generated stable parameter estimates with low FMI (all <0.25 and most <0.10), and concordant results with CCA, indicating a moderate correction for upward bias (Supplementary Data Table S2).

## DISCUSSION

Our main findings were consistent near-target performance across most case management outcomes and, in cases presented at cohort review, appropriate targeting of enhanced support measures for people at risk of non-adherence, along with significantly improved data completeness. Contact identification and assessment fell short of national targets, despite appropriate risk-based targeting. As pre-implementation comparison data were unavailable, the causal effects of cohort review cannot be inferred. Rather, temporal trends in programme performance highlight areas of strength and opportunities for improvement.

The epidemiology of TB in Wales mirrors that of many other European low-incidence countries: disease is increasingly concentrated among migrants from high-incidence countries and in vulnerable and marginalised groups,^3,13^ reflecting a shift from sustained community transmission to reactivation of TB infection.^14^ National control programmes must adapt to these changing epidemiological patterns and employ targeted case finding and screening strategies.^14^ These goals sit within the broader framework of the WHO End TB Strategy to drastically reduce incidence, mortality, and the catastrophic costs of TB globally.^15^ European countries exhibit considerable heterogeneity in the structure of TB control programmes.^16^ Core programme priorities vary, but principally include maintaining political commitment and sustainable funding, strengthening outreach to socially marginalised groups, conducting thorough screening of contacts, ensuring comprehensive data collection and monitoring, and supporting global TB prevention efforts.^4,14^ WHO guidance emphasises that high-quality granular surveillance data are essential for meaningful programme review and for assessing progress towards national targets, particularly as incidence declines.^17,18^

Cohort review operationalises these priorities through a structured governance and quality-improvement mechanism (Figure).^5^ Originally delivered in person, it has transitioned to a more accessible hybrid format as digital tools have been increasingly adopted across health services, particularly since the COVID-19 pandemic.^19^

**Figure.**
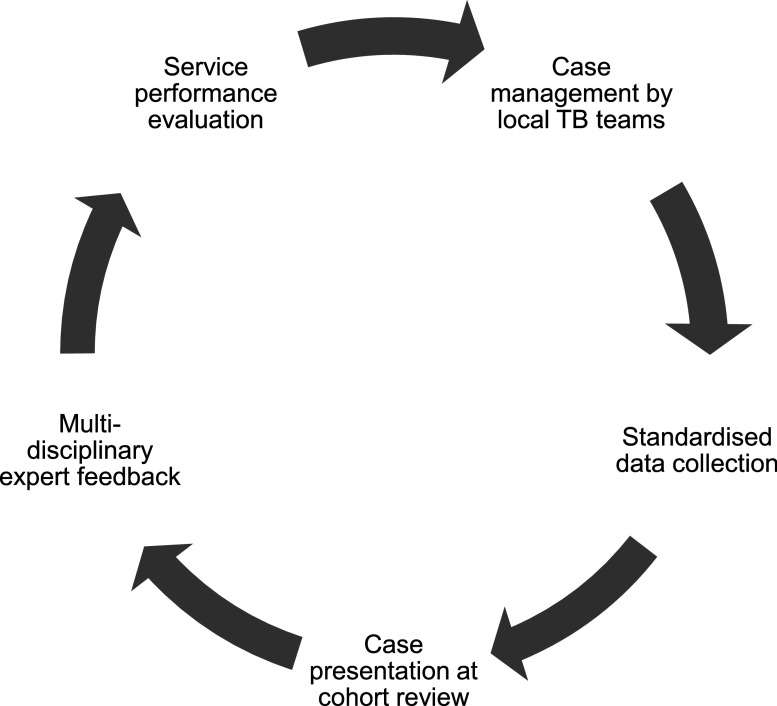
TB cohort review service evaluation cycle.

A key finding was an improvement in the completeness of surveillance data. This likely reflects the preparation required, which incentivises completion and enables real-time correction of missing information. However, programme delivery relies heavily on local nursing capacity. Outside Cardiff and Vale, most nurses do not hold fully dedicated roles, and qualitative findings indicate limited protected time for case preparation and administrative tasks, with cohort review work often undertaken alongside their usual clinical duties.^20^ This provides context for the proportion of cases not presented at cohort review. Some cases were managed in England, particularly in border regions, and presentation was less frequent in health boards without dedicated nursing teams and in earlier programme years, when remote access to meetings was unavailable. Together, these findings suggest that non-presentation for review largely reflected cross-border care pathways and logistical and workforce constraints, rather than the selective omission of complex cases, and emphasise that sustaining robust case-based surveillance in low-incidence settings depends on workforce capacity, with nurses in key coordinating roles.^21^ This mirrors challenges documented in other low-incidence settings, where patient mobility across administrative borders can disrupt TB surveillance and continuity of care.^22^

We also observed increased use of targeted adherence support for cases discussed at cohort review, suggesting that multidisciplinary discussion facilitated earlier recognition of patients at risk of non-adherence and prompted escalation. Qualitative evidence supports this interpretation, characterising cohort review as a reflective learning process that promotes case-based discussion, feedback on management decisions, and support when managing patients with complex needs.^23^ Additionally, nurses in Wales reported that cohort review had led to more individualised management plans and had lowered their threshold for the use of adherence support.^20^ This aligns with elimination frameworks in low-incidence settings that emphasise directing screening and interventions towards high-risk groups, based on local epidemiology.^24^

Contact identification and assessment posed the biggest operational challenge. Index cases not presented at cohort review had lower odds of contact identification, suggesting that structured review reduced operational variance but was insufficient to overcome wider implementation barriers. Systematic identification and clinical assessment of index case contacts, particularly those with smear-positive disease, are a high-yield and cost-effective intervention,^25^ but in low-incidence settings are often constrained by service and patient factors. At a service level, barriers arise primarily during implementation rather than in clinical decision-making, owing to differences in how exposure is defined and investigations are operationalised.^26^ These are the aspects that cohort review is intended to address by standardising practice. However, contact tracing ultimately depends on the participation of individuals who are often asymptomatic and may not perceive themselves as being at risk.^26^ Moreover, barriers such as stigma, unstable housing, and employment difficulties may further reduce willingness to disclose contacts.^27^ Similar barriers were reported by TB teams in Wales.^20^ Consequently, conventional approaches have recognised limitations, and while cohort review may improve reliability, improving outcomes requires complementary approaches, such as collaboration with community outreach organisations that can engage with marginalised groups and act as a bridge to mainstream health care.^28,29^

Increasing case complexity may also have contributed to the modest increase in referral-to-assessment time, as engagement often requires additional coordination, such as arranging interpreter services. Similar social and organisational barriers have been shown to delay access to TB diagnostic services, reflecting implementation challenges rather than clinical prioritisation.^30^ These engagement-related constraints also plausibly explain the observed temporal decline in contact assessment. From 2020 onwards, the trend was further affected by declining health care utilisation and TB service disruptions resulting from the COVID-19 pandemic.^31^ In the UK, 83% of TB services experienced disruption, with almost two thirds reporting specific disruption to contact tracing services, and nearly 70% reporting staff redeployment.^32^ In Wales, redeployment of public health and clinical staff to support screening of Ukrainian refugees may have amplified service-related disruption by limiting capacity.^33^

The primary limitation of this study is its observational design. We cannot conclusively attribute improvements solely to CR implementation without an experimental design or comparison with pre-implementation data. Including historical data would enable a more robust evaluation of the impact of CR, for instance, through an interrupted time-series (ITS) regression analysis.^34^ A further limitation is the incomplete data on key outcome variables in the early years of the study. In a relatively small cohort, reliance on imputed data may have a non-trivial influence on associations and should be considered when interpreting the findings. Additionally, the declining incidence during the study period indicates that resources could be progressively directed towards fewer cases.

As TB incidence declines, progress towards elimination increasingly depends not just on new diagnostics and shorter, better-tolerated treatments, but also on robust and consistent programme delivery. In this study, cohort review acted as a structured quality-assurance mechanism that enhanced surveillance data, supported the appropriate targeting of adherence interventions, and identified ongoing gaps in service delivery. However, the process is resource-intensive, relies heavily on specialist nursing capacity, and is feasible only in settings with manageable case numbers. In such contexts, integrating cohort review into TB programmes may strengthen existing monitoring systems by providing a forum for multidisciplinary interpretation of surveillance data and adjustment to local epidemiology, complementing sustained political engagement and investment as countries work towards elimination.

## Supplementary Material
