# Supplementary material for: National TB cohort review evaluation: insights for control strategies in low-incidence settings
Source: IJTLD Open. 2026 Jun 15;3(6):396–402. doi: 10.5588/ijtldopen.26.0678 (PMC13268076; doi:10.5588/ijtldopen.26.0678)
Supplement: Supplementary file 1 [file ijtldopen26-0678_supplementarydata1.pdf]

Tuberculosis cohort review in Wales: insights for control strategies in low-incidence countries

SUPPLEMENTARY MATERIALS

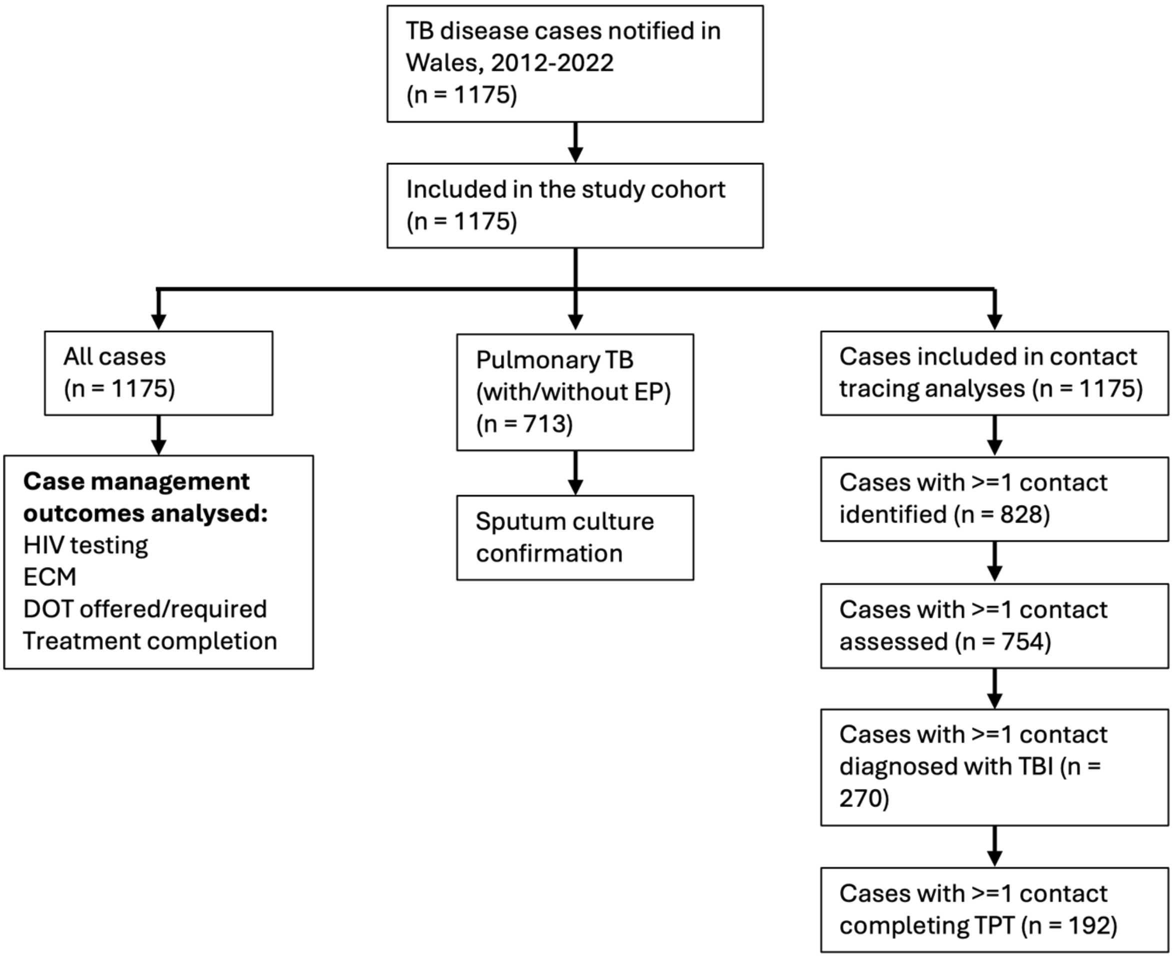

Supplementary Figure S1. Participant flow through the study.

Abbreviations: TB, tuberculosis; HIV, human immunodeficiency virus; ECM, enhanced case management; DOT, directly observed therapy; EP, extra-pulmonary; TBI, tuberculosis infection; TPT, TB preventive treatment.

Supplementary Table S1. Baseline demographic and clinical characteristics of notified TB cases, presented and not presented at cohort review meetings, 2012 to 2022.

	Cohort review status		
**Characteristic**	**Not presented, N = 190 (%)**	**Presented, N = 985 (%)**	**p-value†**
Age, median (IQR)	42 (30-59)	41 (28-58)	0.341
Sex			0.088
Male	128 (17.6)	599 (82.4)	
Female	62 (13.8)	386 (86.2)	
Migration status			0.235
UK-born	81 (17.1)	392 (82.9)	
Non-UK-born	101 (14.6)	593 (85.4)	
Social risk factors, n/N (%)*			
≥1	20/169 (11.8)	145/960 (15.1)	0.267
≥2	10/169 (5.9)	80/960 (8.3)	0.285
- Missing (any)	64/190 (33.7)	139/985 (14.1)	**<0.001**
Local health board			**<0.001**
Aneurin Bevan	18 (7.1)	236 (92.9)	
Betsi Cadwaladr	8 (4.4)	174 (95.6)	
Cardiff and Vale	13 (3.8)	333 (96.2)	
Cwm Taf Morgannwg	37 (32.7)	76 (67.3)	
Hywel Dda	24 (24.2)	75 (75.8)	
Swansea Bay	39 (30.0)	91 (70.0)	
Outside Wales	51 (100.0)	0 (0.0)	
Calendar period			**<0.001**
2012-2015	112 (21.3)	413 (78.7)	
2016-2018	31 (10.0)	280 (90.0)	
2019-2022	47 (13.9)	292 (86.1)	
TB site			0.710
PTB with/without EPTB	113 (15.8)	600 (84.2)	
Extra-pulmonary only	77 (16.7)	385 (83.3)	

Descriptive characteristics of notified TB cases in Wales, comparing cases presented and not presented at cohort review meetings. Complete-case analysis data were used. Where missingness was absent or <5%, it was omitted from the table for parsimony. Significant p-values (p<0.05) are shown in bold.

* Social risk factors include a history of homelessness, prison, substance use disorders and alcohol misuse. Missing data is reported at the field level across the 4 components.

† p-values are derived from Cochran-Armitage tests for categorical data with binary outcomes across ordinal time periods, non-ordered categorical variables using chi squared, and Kruskal-Wallis test for continuous data.

Abbreviations: TB, tuberculosis; CR, cohort review; IQR, interquartile range; UK, United Kingdom; PTB, pulmonary tuberculosis; EPTB, extra-pulmonary tuberculosis; MDR, multidrug-resistant; DOT, directly observed therapy; TPT, TB preventive treatment.

Supplementary Table S2a. Case management outcomes, with sensitivity analyses of multiple imputation and complete-case analysis, for notified TB cases in Wales, 2012 to 2022.

Variable	Contrast	MI unadj. OR (95% CI), p	MI adj. OR (95% CI), p	FMI	MI adj. exp. OR (95% CI), p	FMI	Diff. OR (%)	CCA unadj. OR (95% CI), p	CCA adj. OR (95% CI), p
**HIV**			30 vars; m=20		34 vars; m=40				
Year of notification	Per 1-year increase	**1.28 (1.17-1.40), <0.001**	**1.28 (1.17-1.41), <0.001**	0.003	**1.28 (1.17-1.41), p<0.001**	0.006	<0.1	**1.27 (1.17-1.40), <0.001**	**1.29 (1.17-1.41), <0.001**
Year x Not presented	Interaction term	0.99 (0.75-1.30), 0.919	1.01 (0.75-1.35), 0.954	0.002	1.01 (0.75-1.35), 0.953	0.003	<0.1	0.98 (0.75-1.29), 0.911	1.01 (0.75-1.35), 0.961
Presented	Not presented vs presented		0.60 (0.27-1.29), 0.190	0.002	0.60 (0.27-1.28), 0.191	0.003	<0.1		0.60 (0.27-1.27), 0.192
Age	Per 10 years		Spline	0.004	Spline	0.003	<0.1		Spline
Sex	Male vs female		1.15 (0.70-1.89), 0.588	0.004	1.15 (0.70-1.89), 0.589	0.005	<0.1		1.14 (0.69-1.90), 0.601
Any social risk factor	Yes vs no		2.37 (0.97-5.79), 0.060	0.002	2.36 (0.97-5.79), 0.060	0.002	<0.1		2.37 (0.97-5.8), 0.058
UK-born	Non-UK-born vs UK-born		**1.98 (1.14-3.44), 0.014**	0.007	**1.99 (1.15-3.46), 0.014**	0.004	0.5		**2.00 (1.16-3.47), 0.013**
TB site	Pulmonary ± EP vs EP only		0.61 (0.36-1.05), 0.076	0.005	0.62 (0.36-1.06), 0.083	0.006	1.0		0.61 (0.36-1.05), 0.077
**ECM**									
Year of notification	Per 1-year increase	1.05 (1.00-1.09), 0.057	1.03 (0.98-1.08), 0.251	0.061	1.03 (0.98-1.08), 0.283	0.070	<0.1	**1.06 (1.01-1.11), 0.015**	1.05 (0.99-1.10), 0.073
Year x Not presented	Interaction term	0.90 (0.72-1.13), 0.360	0.94 (0.73-1.20), 0.623	0.072	0.95 (0.75-1.21), 0.676	0.044	1.1	0.83 (0.66-1.05), 0.116	0.87 (0.68-1.13), 0.310
Presented	Not presented vs presented		**0.36 (0.17-0.78), 0.010**	0.072	**0.36 (0.17-0.77), 0.008**	0.044	<0.1		**0.32 (0.15-0.71), 0.004**
Age	Per 10 years		0.92 (0.85-1.00), 0.050	0.099	**0.91 (0.84-0.99), 0.029**	0.078	-1.1		0.93 (0.85-1.01), 0.078
Sex	Male vs female		0.94 (0.70-1.26), 0.666	0.030	0.95 (0.71-1.29), 0.753	0.040	1.1		0.93 (0.68-1.26), 0.648
Any social risk factor	Yes vs no		**5.83 (3.54-9.59), <0.001**	0.042	**5.71 (3.47-9.40), <0.001**	0.036	-2.1		**6.15 (3.70-10.2), <0.001**
UK-born	Non-UK-born vs UK-born		0.84 (0.60-1.17), 0.308	0.075	0.82 (0.59-1.14), 0.241	0.032	-2.4		0.91 (0.65-1.28), 0.586
TB site	Pulmonary ± EP vs EP only		**2.37 (1.73-3.24), <0.001**	0.050	**2.38 (1.74-3.24), <0.001**	0.029	0.4		**2.49 (1.81-3.42), <0.001**
**DOT required**									
Year of notification	Per 1-year increase	**1.10 (1.05-1.17), <0.001**	**1.09 (1.02-1.16), 0.012**	0.136	**1.07 (1.01-1.15), 0.043**	0.202	-1.8	**1.12 (1.07-1.18), <0.001**	**1.12 (1.05-1.19), 0.001**
Year x Not presented	Interaction term	**0.75 (0.61-0.93), 0.007**	**0.75 (0.58-0.97), 0.027**	0.030	**0.74 (0.57-0.97), 0.027**	0.044	-1.3	**0.71 (0.57-0.88), 0.002**	**0.71 (0.54-0.93), 0.012**
Presented	Not presented vs presented		0.91 (0.44-1.88), 0.791	0.030	0.86 (0.41-1.81), 0.690	0.044	-5.5		UE
Age	Per 10 years		Spline	0.039	Spline	0.061	-		Spline
Sex	Male vs female		1.25 (0.83-1.86), 0.281	0.053	1.27 (0.85-1.88), 0.238	0.033	1.6		1.23 (0.81-1.86), 0.315
Any social risk factor	Yes vs no		**11.81 (7.48-18.64), <0.001**	0.031	**11.42 (7.10-17.74), <0.001**	0.050	-3.3		**12.7 (8.02-20.3), <0.001**
UK-born	Non-UK-born vs UK-born		0.70 (0.47-1.05), 0.083	0.056	0.68 (0.45-1.01), 0.058	0.068	-2.9		0.72 (0.48-1.08), 0.114
TB site	Pulmonary ± EP vs EP only		**2.29 (1.48-3.53), <0.001**	0.042	**2.31 (1.50-3.58), <0.001**	0.056	0.9		**2.60 (1.65-4.08), <0.001**
**DOT offered**									
Year of notification	Per 1-year increase	1.28 (0.94-1.75), 0.122	1.24 (0.90-1.72), 0.187	0.133	1.22 (0.87-1.70), 0.253	0.215	-1.6	**1.45 (1.03-2.05), 0.029**	1.37 (0.97-1.93), 0.070
Year x Not presented	Interaction term	0.78 (0.41-1.49), 0.453	0.81 (0.42-1.57), 0.531	0.024	0.82 (0.42-1.60), 0.564	0.018	1.2	0.61 (0.30-1.24), 0.180	0.65 (0.32-1.31), 0.226
Presented	Not presented vs presented		**0.14 (0.02-0.92), 0.041**	0.024	**0.13 (0.02-0.86), 0.035**	0.018	-7.1		0.17 (0.02-1.26), 0.084
Age	Per 10 years		1.00 (0.62-1.62), 0.998	0.018	0.99 (0.61-1.61), 0.981	0.025	-1.0		1.01 (0.62-1.64), 0.963
Sex	Male vs female		1.15 (0.15-8.79), 0.892	0.052	1.16 (0.15-8.97), 0.883	0.074	0.9		1.50 (0.19-12.06), 0.700
Any social risk factor	Yes vs no		1.36 (0.15-12.39), 0.784	0.101	1.36 (0.14-12.78), 0.789	0.124	<0.1		1.12 (0.12-10.10), 0.920
UK-born	Non-UK-born vs UK-born		0.36 (0.05-2.39), 0.288	0.093	0.37 (0.06-2.66), 0.337	0.065	2.7		3.60 (0.05-2.62), 0.313
TB site	Pulmonary ± EP vs EP only		1.40 (0.16-12.20), 0.760	0.039	1.40 (0.16-12.16), 0.756	0.068	<0.1		2.14 (0.23-20.01), 0.506

Odds ratios (OR) and 95% confidence intervals (95% CI) were derived from logistic regression models. Adjusted models include age, sex, migration status, the presence of ≥1 social risk factor (homelessness, prison, and substance or alcohol misuse), pulmonary vs extra-pulmonary TB (TB site). Interaction terms for Year x Presented denote whether there was a difference in temporal trend in cases not presented vs presented at cohort review meetings. Multiple imputation by chained equations was used to handle missing data (m=40). Results were robust to changes in imputation specification and complete case sensitivity analysis. Sputum culture was not imputed due to the proportion of missing information. Abbreviations: Adj, adjusted; Unadj, unadjusted; MI, multiple imputation; FMI, fraction of missing information; CCA, complete-case analysis; exp, expanded; UK, United Kingdom; ECM, enhanced case management; DOT, directly observed therapy; EP, extra-pulmonary; UE, unable to estimate.

Supplementary Table S2b. Case management outcomes, with sensitivity analyses of multiple imputation and complete-case analysis, for notified TB cases in Wales, 2012 to 2022.

Variable	Contrast	MI unadj. OR (95% CI), p	MI adj. OR (95% CI), p	FMI	MI adj. exp. OR (95% CI), p	FMI	Diff. OR (%)	CCA unadj. OR (95% CI), p	CCA adj. OR (95% CI), p
**Sputum culture**									
Year of notification	Per 1-year increase	1.02 (0.93-1.12), 0.673	1.00 (0.90-1.11), 0.957	0.197	1.02 (0.91-1.13), 0.772	0.197	2.0	0.95 (0.85-1.07), 0.480	0.90 (0.73-1.12), 0.321
Year x Not presented	Interaction term	1.11 (0.54-2.28), 0.766	1.09 (0.54-2.19), 0.813	0.165	1.15 (0.57-2.33), 0.698	0.164	5.5	0.51 (0.21-1.26), 0.145	1.11 (0.98-1.26), 0.710
Presented	Not presented vs presented		5.24 (0.76-37.75), 0.997	0.176	4.36 (0.63-30.02), 0.136	0.239	-7.3		UE
Age	Per 10 years		Spline	0.275	Spline	0.268	-		Spline
Sex	Male vs female		1.27 (0.71-2.26), 0.414	0.172	1.20 (0.62-1.99), 0.723	0.152	-5.5		1.41 (0.65-3.03), 0.384
Any social risk factor	Yes vs no		**2.78 (1.19-6.50), 0.018**	0.183	**2.74 (1.48-5.08), 0.001**	0.190	-1.4		**2.96 (1.09-8.03), 0.033**
UK-born	Non-UK-born vs UK-born		**2.25 (1.25-4.06), 0.007**	0.146	**2.35 (1.16-5.82), 0.020**	0.138	4.4		**3.79 (1.69-8.45), 0.001**
TB site	Pulmonary ± EP vs EP only								-
**Treatment completion**									
Year of notification	Per 1-year increase	1.05 (0.99-1.11), 0.112	1.05 (0.99-1.11), 0.106	0.013	1.06 (0.99-1.12), 0.073	0.016	0.9	1.05 (1.00-1.16), 0.070	1.05 (0.99-1.12), 0.064
Year x Not presented	Interaction term	0.98 (0.84-1.13), 0.744	0.94 (0.80-1.10), 0.454	0.017	0.96 (0.82-1.14), 0.663	0.023	2.1	1.01 (0.87-1.18), 0.886	0.97 (0.83-1.15), 0.774
Presented	Not presented vs presented		1.00 (0.60-1.65), 0.986	0.017	1.04 (0.62-1.74), 0.880	0.023	4.0		1.02 (0.76-5.90), 0.774
Age	Per 10 years		Spline	0.004	Spline	0.004	-		Spline
Sex	Male vs female		0.79 (0.54-1.17), 0.240	0.004	0.81 (0.55-1.18), 0.269	0.003	2.5		0.81 (0.55-1.19), 0.277
Any social risk factor	Yes vs no		0.60 (0.36-1.00), 0.052	0.007	0.60 (0.36-1.01), 0.563	0.010	<0.1		0.61 (0.36-1.03), 0.066
UK-born	Non-UK-born vs UK-born		0.85 (0.56-1.28), 0.434	0.006	0.86 (0.57-1.30), 0.466	0.004	1.2		0.85 (0.57-1.30), 0.234
TB site	Pulmonary ± EP vs EP only		0.78 (0.52-1.15), 0.208	0.005	0.79 (0.53-1.17), 0.234	0.003	1.3		0.98 (0.53-1.17), 0.234

Odds ratios (OR) and 95% confidence intervals (95% CI) were derived from logistic regression models. Adjusted models include age, sex, migration status, the presence of ≥1 social risk factor (homelessness, prison, and substance or alcohol misuse), pulmonary vs extra-pulmonary TB (TB site). Interaction terms for Year x Presented denote whether there was a difference in temporal trend in cases not presented vs presented at cohort review meetings. Multiple imputation by chained equations was used to handle missing data (m=40). Results were robust to changes in imputation specification and complete case sensitivity analysis. Sputum culture was not imputed due to the proportion of missing information. Abbreviations: Adj, adjusted; Unadj, unadjusted; MI, multiple imputation; FMI, fraction of missing information; CCA, complete-case analysis; exp, expanded; UK, United Kingdom; ECM, enhanced case management; DOT, directly observed therapy; EP, extra-pulmonary; UE, unable to estimate.

Supplementary Figure S2. Adjusted natural cubic spline relationships between age and TB case management outcomes

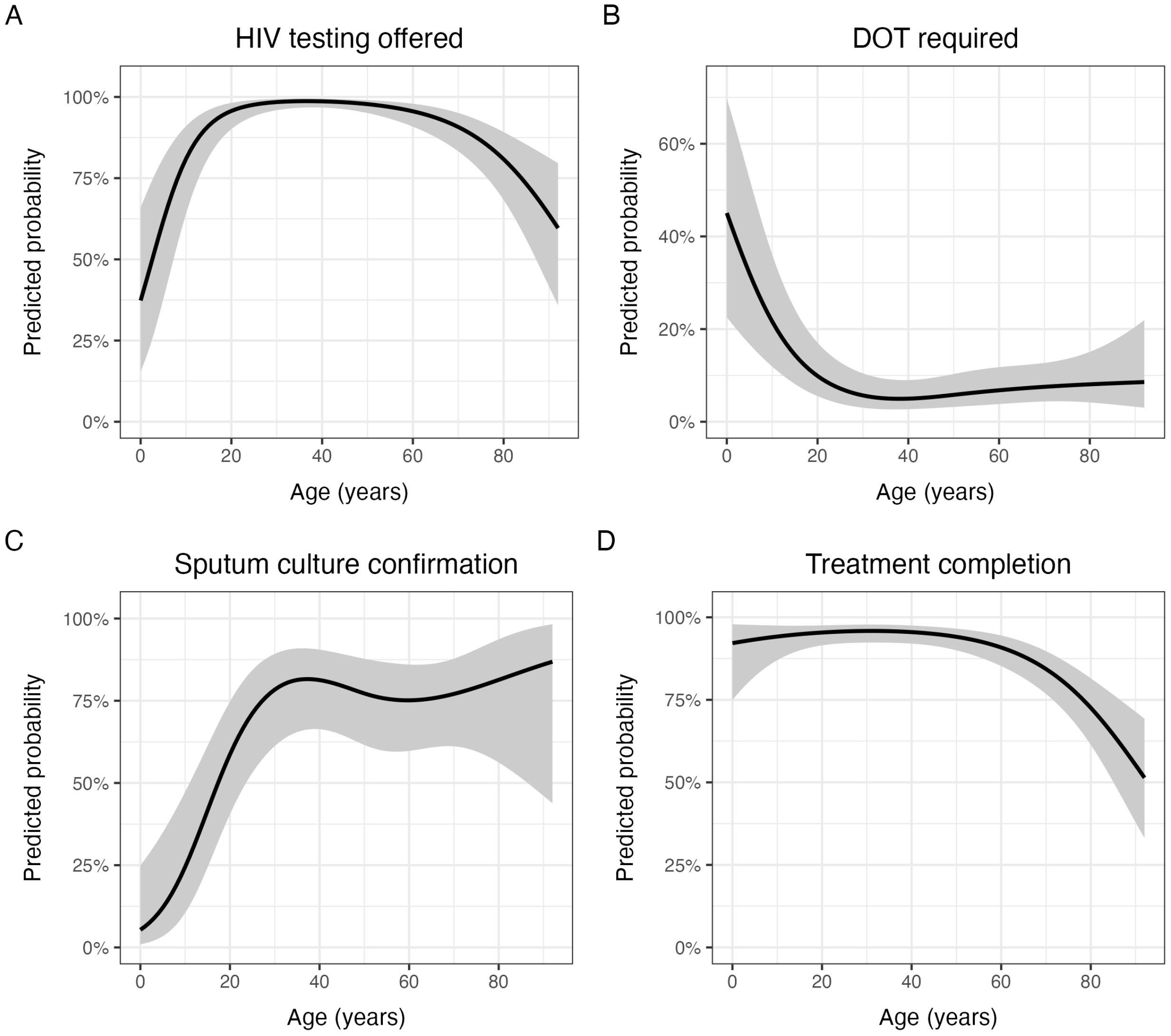

Predicted probabilities derived from multivariable logistic regression models with age modeled as a natural cubic spline (3 degrees of freedom; default quantile-based knot placement). Models adjusted for year, sex, ≥1 social risk factors,migration status, and TB site. Estimates pooled across 40 multiply imputed datasets. Curves show adjusted predicted probabilities with 95% confidence intervals (shaded regions).

Supplementary Table S3. Contact-tracing outcomes, with sensitivity analyses using multiple imputation specifications and complete-case analysis, for notified TB cases in Wales, 2012 to 2022.

Variable	Contrast	MI unadj. effect (95% CI), p	MI adj. effect (95% CI), p	FMI	MI adj. exp. effect (95% CI), p	FMI	Diff. OR (%)
**Contacts identified (IRR)**			30 vars; m=20		34 vars; m=40		
Year of notification	Per 1-year increase	Non-linear, LRT p<0.001	Non-linear, LRT p<0.001	0.002	Non-linear, LRT p<0.001	0.002	<0.1
Year x Not presented	Interaction term	0.99 (0.92-1.07), 0.758	1.00 (0.93-1.08), 0.901	0.002	0.99 (0.92-1.07), 0.758	0.002	<0.1
Presented	Not presented vs pres		**0.28 (0.22-0.36), <0.001**	0.002	**0.28 (0.22-0.36), <0.001**	0.002	<0.1
Age	Per 10 years		0.98 (0.94-1.03), 0.443	0.002	0.98 (0.94-1.03), 0.443	0.002	<0.1
Sex	Male vs female		**0.73 (0.62-0.86), p<0.001**	0.002	**0.73 (0.62-0.86), <0.001**	0.002	<0.1
Any social risk factor	Yes vs no		1.26 (0.99-1.59), 0.059	0.002	1.26 (0.99-1.59), 0.059	0.002	<0.1
UK-born	Non-UK-born vs UK-born		**0.82 (0.68-0.98), 0.028**	0.002	**0.82 (0.68-0.98), 0.028**	0.002	<0.1
TB site	Pulmonary ± EP vs EP only		**2.73 (2.29-3.25), <0.001**	0.002	**2.73 (2.29-3.25), <0.001**	0.002	<0.1
Core + cavities	Cavities present		**1.39 (1.13-1.71), 0.002**	0.002	**1.39 (1.13-1.71), 0.002**	0.002	<0.1
Core + smear positive	Smear positive		**1.76 (1.43-2.17), <0.001**	0.002	**1.76 (1.43-2.17), <0.001**	0.002	<0.1
**Contacts assessed**							
Year of notification	Per 1-year increase	Non-linear, LRT p<0.001	Non-linear, LRT p<0.001	0.002	Non-linear, LRT p<0.001	0.002	<0.1
Year x Not presented	Interaction term	1.07 (0.87-1.33), 0.515	1.08 (0.86-1.35), 0.525	0.002	1.08 (0.86-1.35), 0.525	0.002	<0.1
Presented	Not presented vs pres		1.39 (0.68-2.86), 0.368	0.002	1.39 (0.68-2.87), 0.367	0.002	<0.1
Age	Per 10 years		**0.91 (0.84-0.98), 0.009**	0.002	**0.91 (0.84-0.98), 0.009**	0.002	<0.1
Sex	Male vs female		**1.36 (1.03-1.80), 0.032**	0.002	**1.36 (1.03-1.80), 0.037**	0.002	<0.1
Any social risk factor	Yes vs no		**0.55 (0.40-0.78), <0.001**	0.002	**0.55 (0.40-0.78), 0.001**	0.002	<0.1
UK-born	Non-UK-born vs UK-born		1.16 (0.86–1.56), 0.337	0.002	1.16 (0.86-1.56), 0.338	0.002	<0.1
TB site	Pulmonary ± EP vs EP only		0.95 (0.64-1.42), 0.809	0.002	**0.95 (0.64-1.42), 0.809**	0.002	<0.1
Core + cavities	Cavities present		**2.49 (1.67-3.71), <0.001**	0.002	**2.49 (1.67-3.71), <0.001**	0.002	<0.1
Core + smear positive	Smear positive		0.65 (0.42-1.00), 0.051	0.002	0.65 (0.42-1.00), 0.051	0.002	<0.1
**TPT completion**							
Year of notification	Per 1-year increase	1.05 (0.98-1.14), 0.186	1.03 (0.95-1.11), 0.474	0.010	1.05 (0.96-1.15), 0.269	0.010	<0.1
Year x Not presented	Interaction term	1.30 (0.93-1.82), 0.123	1.30 (0.93-1.81), 0.124	0.010	1.26 (0.90-1.75), 0.178	0.010	<0.1
Presented	Not presented vs pres		0.71 (0.26-1.91), 0.498	0.010	0.71 (0.26-1.92), 0.499	0.010	<0.1
Age	Per 10 years		0.93 (0.79–1.08), 0.320	0.010	0.93 (0.79–1.05), 0.320	0.010	<0.1
Sex	Male vs female		**0.50 (0.28–0.87), 0.014**	0.010	**0.50 (0.28–0.87), 0.014**	0.010	<0.1
Any social risk factor	Yes vs no		0.86 (0.46–1.60), 0.622	0.010	0.86 (0.46–1.60), 0.622	0.010	<0.1
UK-born	Non-UK-born vs UK-born		0.40 (0.14–1.13), 0.082	0.010	0.40 (0.14–1.13), 0.082	0.010	<0.1
TB site	Pulmonary ± EP vs EP only		0.74 (0.43–1.28), 0.280	0.010	0.74 (0.43–1.28), 0.280	0.010	<0.1
Core + cavities	Cavities present		0.60 (0.32–1.11), 0.101	0.010	0.60 (0.32–1.11), 0.101	0.010	<0.1
Core + smear positive	Smear positive		**2.17 (1.11–4.22), 0.024**	0.010	**2.17 (1.12–4.22), 0.024**	0.010	<0.1

Incident rate ratios (IRR) and odds ratios (OR) with 95% confidence intervals (CI). Contact identification analysed using negative binomial regression. Contact assessment and TPT completion analysed using quasibinomial logistic regression. Multiple imputation was applied to predictor variables only; outcome variables were not imputed. Analyses therefore included all cases with observed contact tracing outcomes using imputed predictor data. Year and age tested for linearity using likelihood-ratio tests comparing linear and spline terms and presented with natural cubic splines (3df) where non-linearity detected. Contact assessment limited to cases with ≥1 contact identified (n=828) and TPT completion to cases with ≥1 contact diagnosed with TB infection (n=270). Extended models show effect estimates when CXR cavities or sputum smear positivity were added to the models. Interaction terms are shown as linear for parsimony and indicate the difference in trend between cases not presented vs presented at TB cohort review. Any social risk factor refers to ≥1 self-reported risk factor (history of homelessness, prison, and substance or alcohol misuse). FMI indicates fraction of missing information, reflecting uncertainty in coefficient estimates due to missing predictor data. Sensitivity analyses compared original (30 auxiliary variables, m=20) and expanded (34 auxiliary variables, m=40) imputation specifications.

Supplementary Figure S3. Adjusted natural cubic splines for contact tracing outcomes

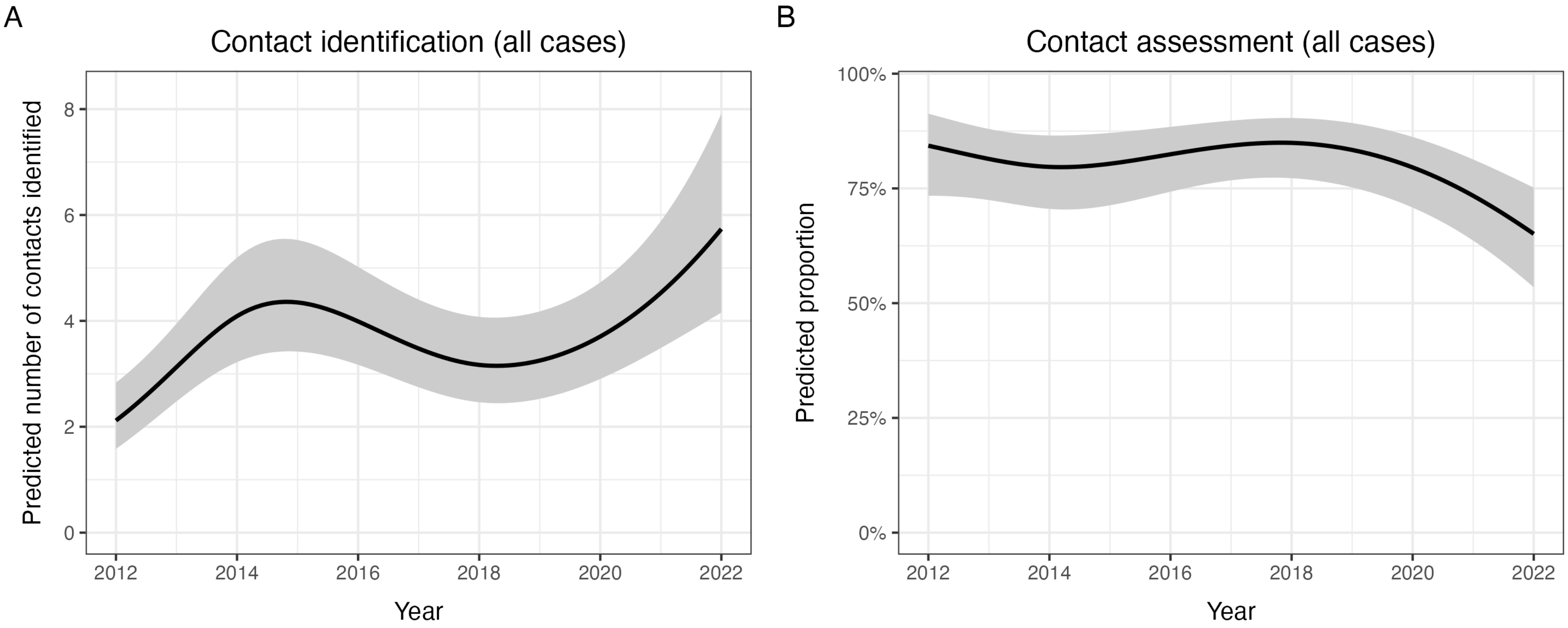

Predicted number of contacts identified (Panel A) and predicted proportion of contacts assessed (Panel B) for all notified TB cases. Contact identification analysed using negative binomial regression to account for overdispersion in count data. Contact assessment analysed using quasibinomial regression with explicit denominators. Year modeled as natural cubic spline (3 degrees of freedom; default quantile-based knot placement) after linearity rejected by likelihood ratio tests. Models adjusted for age, sex, ≥1 social risk factors, migration status, and TB site. Estimates pooled across 40 multiply imputed datasets. Curves show adjusted predictions with 95% confidence intervals (shaded regions).

Supplementary Table S4. Missing data by time period and overall for notified TB cases, with predictors of missing data, 2012 to 2022.

	Missing, n/N (%)				Predictors
**Demographics**	**2012 – 2015**	**2016 – 2018**	**2019 - 2022**	**2012 – 2022**	
Year case notified	Complete	Complete	Complete	Complete	-
Age	Complete	Complete	Complete	Complete	-
Male sex	Complete	Complete	Complete	Complete	-
Social risk factors*	222/2100 (10.6)	102/1244 (8.2)	95/1356 (7.0)	419/4700 (8.9)	-
Migration status	6/525 (1.1)	2/311 (0.6)	Complete	8/1175 (0.7)	-
UK entry year	19/307 (6.2)	9/164 (5.5)	4/223 (1.8)	123/1175 (10.5)	-
Local health board	Complete	Complete	Complete	Complete	-
**Clinical**					
PTB vs EPTB	3/525 (0.6)	Complete	1/339 (0.3)	4/1175 (0.3)	-
Sputum culture	280/292 (95.9)	72/204 (35.3)	47/217 (21.7)	399/713 (56)	Year (OR 0.59, p<0.001), PTB vs EPTB (0.11, p<0.001).
Sputum smear	77/292 (26.4)	48/204 (23.5)	57/217 (26.3)	182/713 (25.5)	Not presented (OR 3.96, p<0.001), PTB vs EPTB (0.09, p<0.001), BC (1.61, p=0.042).
CXR findings	134/525 (25.5)	32/311 (10.3)	106/339 (31.3)	272/1175 (23.1)	Year (OR 1.09, p=0.003), Not presented (15.62, p<0.001), UK-born (0.62, p=0.034), BC (1.75, p=0.047), HDD (3.2, p<0.001).
MDR	94/525 (17.9)	28/311 (9.0)	35/339 (10.3)	157/1175 (13.4)	-
**Case management**					
Presented	Complete	Complete	Complete	Complete	-
HIV testing	111/525 (21.2)	14/311 (4.5)	13/339 (3.8)	138/1175 (11.7)	Year (OR 0.40, p<0.001), Not presented (11.6, p<0.001), strongly predicted by LHBs (BC, CTM, HDD, and SB; OR range 2.4-7.9; p<0.001).
ECM	224/525 (42.7)	53/311 (17.0)	56/339 (16.5)	333/1175 (28.3)	Year (OR 0.71, p<0.001), Not presented (8.49, p<0.001), Male sex (1.47, p=0.029), strongly predicted by LHBs (BC, CTM, HDD, SB and outside Wales; OR range 1.5-9.45; p<0.001).
DOT required	176/525 (33.5)	48/311 (15.4)	25/339 (7.4)	249/1175 (21.2)	Year (OR 0.68, p<0.001), Not presented (7.18, p<0.001), UK-born (0.61, p=0.029), strongly predicted by LHBs (BC, CTM, HDD, and SB; OR range 1.6-69.0; p<0.001).
DOT offered	9/50 (18.0)	6/62 (9.7)	2/89 (2.2)	17/201 (8.5)	Year (OR 0.76, p<0.001), Not presented (6.0, p<0.001), strongly predicted by LHBs (BC, CTM, HDD, and SB; OR range 1.6-69.0; p<0.001).
Treatment completion	17/525 (3.2)	7/311 (2.3)	11/339 (3.2)	35/1175 (3.0)	Not presented (OR 2.53, p=0.025), PTB vs EPTB (2.77, p=0.037), CTM (8.86, p=0.09), SB (14.19, p<0.001).

Data is derived from complete case analysis. Predictors were derived by fitting logistic regression models with year, age, sex, local health board, social risk factors, TB site, and migration status as predictors. Across all models, overall fit was strong, with likelihood ratio χ^2 tests highly significant (all p<0.001) and good to excellent discrimination (area under the curve range 0.81-0.97), indicating that missingness was strongly associated with observed covariates.

* Social risk factors include a history of homelessness, prison, substance use disorders and alcohol misuse. Missing data is reported at the field level across the 4 components.

Abbreviations: TB, tuberculosis; CR, cohort review; IQR, interquartile range; UK, United Kingdom; PTB, pulmonary tuberculosis; EPTB, extra-pulmonary tuberculosis; MDR, multidrug-resistant; DOT, directly observed therapy; TPT, TB preventive treatment.

Supplementary Table S5. Temporal trends in data completeness for key tuberculosis cohort review outcome variables in Wales, 2012 to 2022.

Variable	Recorded 2012, n/N (%)	Recorded 2022, n/N (%)	pp change recorded	OR per year recorded (95% CI), p	OR trend per year not presented (95% CI), p
Overall	324/656 (49.4)	312/342 (91.2)	41.8	1.29 (1.26-1.32), <0.001	0.89 (0.85-0.93), <0.001
HIV	91/143 (63.6)	68/71 (95.8)	32.1	1.43 (1.32-1.55), <0.001	0.50 (0.35-0.71), <0.001
ECM	34/143 (23.8)	61/71 (85.9)	62.1	1.32 (1.25-1.38), <0.001	0.88 (0.77-1.00), 0.053
DOT required	58/143 (40.6)	66/71 (93.0)	52.4	1.36 (1.28-1.44), <0.001	0.79 (0.69-0.91), <0.001
DOT offered	4/7 (57.1)	17/18 (94.4)	37.3	1.24 (1.18-1.29), <0.001	0.86 (0.77-0.97), 0.017
Sputum culture	1/77 (1.3)	32/40 (80.0)	78.7	1.55 (1.47-1.64), <0.001	0.75 (0.65-0.85), <0.001
Treatment completion	136/143 (95.1)	68/71 (95.8)	0.7	1.04 (0.93-1.16), 0.520	0.90 (0.73-1.12), 0.343

Temporal trends in data completeness for key TB surveillance variables. Odds ratios (OR) were estimated using logistic regression with year as a continuous variable. OR trend per year not recorded shows the annual change stratified by presentation of cases at TB cohort review.
Abbreviations: ECM, enhanced case management; DOT, directly observed therapy; pp, percentage point change; OR, odds ratio; CI, confidence interval.
